# 3-(4-Methoxybenzyl)-1,5-benzo­thiazepin-4(5*H*)-one

**DOI:** 10.1107/S1600536813009215

**Published:** 2013-04-10

**Authors:** R. Selvakumar, M. Bakthadoss, S. Vijayakumar, S. Murugavel

**Affiliations:** a Department of Organic Chemistry, University of Madras, Maraimalai Campus, Chennai 600 025, India; b Department of Chemistry, Pondicherry University, Puducherry 605 014, India; c Department of Physics, Sri Balaji Chokkalingam Engineering College, Arni, Thiruvannamalai 632 317, India; d Department of Physics, Thanthai Periyar Government Institute of Technology, Vellore 632 002, India

## Abstract

In the title compound, C_17_H_15_NO_2_S, the thia­zepine ring adopts a slightly distorted twist-boat conformation. The dihedral angle between the mean plane of the benzo­thia­zepin ring system and the benzene ring is 65.7 (1)°. In the crystal, pairs of N—H⋯O hydrogen bonds link inversion-related mol­ecules into dimers, generating *R_2_^2^*(8) ring motifs. These dimers are further linked by C—H⋯π and π–π inter­actions [inter-centroid distance between the benzene rings of the benzo­thia­zepine unit = 3.656 (3) Å] into a three-dimensional supra­molecular network.

## Related literature

For background to the biology of thia­zepin derivatives and for a related structure, see: Bakthadoss *et al.* (2013 ▶). For ring-puckering parameters, see: Cremer & Pople (1975 ▶).
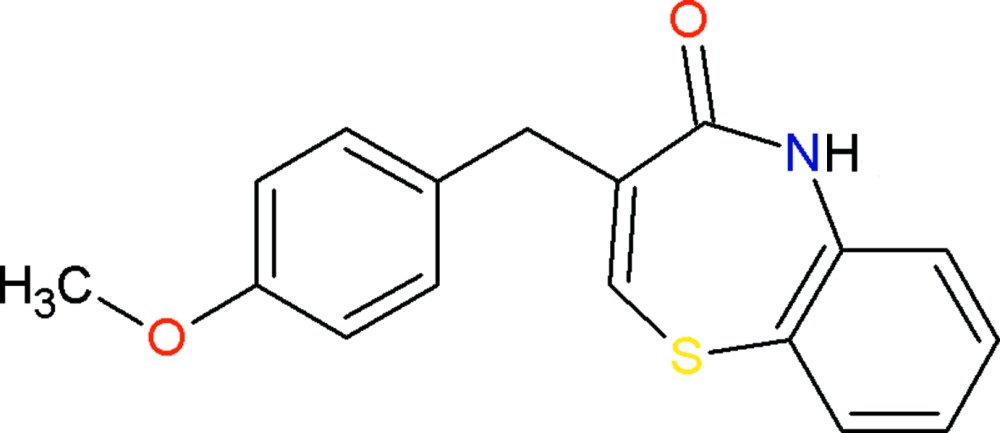


## Experimental

### 

#### Crystal data

C_17_H_15_NO_2_S
*M* *_r_* = 297.36
Triclinic, 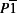

*a* = 7.678 (5) Å
*b* = 9.612 (5) Å
*c* = 10.860 (5) Å
α = 77.208 (5)°
β = 74.117 (4)°
γ = 81.522 (5)°
*V* = 748.5 (7) Å^3^
*Z* = 2
Mo *K*α radiation
μ = 0.22 mm^−1^
*T* = 293 K
0.23 × 0.21 × 0.15 mm

#### Data collection

Bruker APEXII CCD diffractometer
Absorption correction: multi-scan (*SADABS*; Sheldrick, 1996 ▶) *T*_min_ = 0.951, *T*_max_ = 0.968
18449 measured reflections
5363 independent reflections
3676 reflections with *I* > 2σ(*I*)
*R*_int_ = 0.027

#### Refinement

*R*[*F* ^2^ > 2σ(*F* ^2^)] = 0.045
*wR*(*F* ^2^) = 0.134
*S* = 1.05
5363 reflections
191 parameters
H-atom parameters constrained
Δρ_max_ = 0.28 e Å^−3^
Δρ_min_ = −0.33 e Å^−3^

### 

Data collection: *APEX2* (Bruker, 2004 ▶); cell refinement: *APEX2* and *SAINT* (Bruker, 2004 ▶); data reduction: *SAINT* and *XPREP* (Bruker, 2004 ▶); program(s) used to solve structure: *SHELXS97* (Sheldrick, 2008 ▶); program(s) used to refine structure: *SHELXL97* (Sheldrick, 2008 ▶); molecular graphics: *ORTEP-3 for Windows* (Farrugia, 2012) ▶; software used to prepare material for publication: *SHELXL97* and *PLATON* (Spek, 2009 ▶).

## Supplementary Material

Crystal structure: contains datablock(s) global, I. DOI: 10.1107/S1600536813009215/sj5315sup1.cif

Structure factors: contains datablock(s) I. DOI: 10.1107/S1600536813009215/sj5315Isup2.hkl

Supplementary material file. DOI: 10.1107/S1600536813009215/sj5315Isup3.cml

Additional supplementary materials:  crystallographic information; 3D view; checkCIF report

## Figures and Tables

**Table 1:** Hydrogen-bond geometry (Å, °) *Cg* is the centroid of the C3–C7 benzene ring.

*D*—H⋯*A*	*D*—H	H⋯*A*	*D*⋯*A*	*D*—H⋯*A*
N1—H1*A*⋯O1^i^	0.86	2.02	2.860 (2)	167
C17—H17*B*⋯*Cg* ^ii^	0.96	2.96	3.561 (3)	122

Symmetry codes: (i) 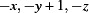; (ii) 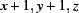.

**Fig. 1.**
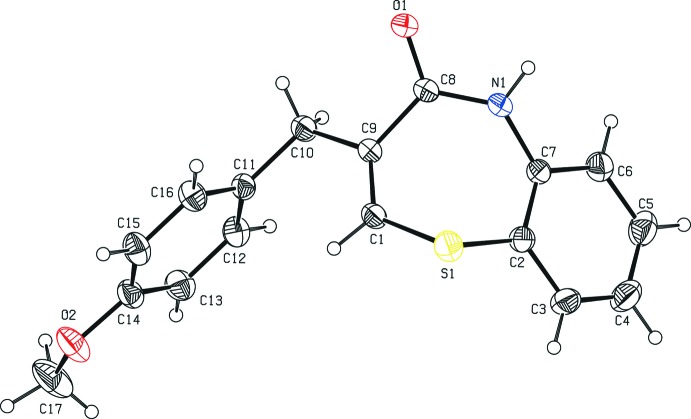
Molecular structure of the title compound showing displacement ellipsoids at the 30% probability level. H atoms are presented as a small spheres of arbitrary radii.

**Fig. 2.**
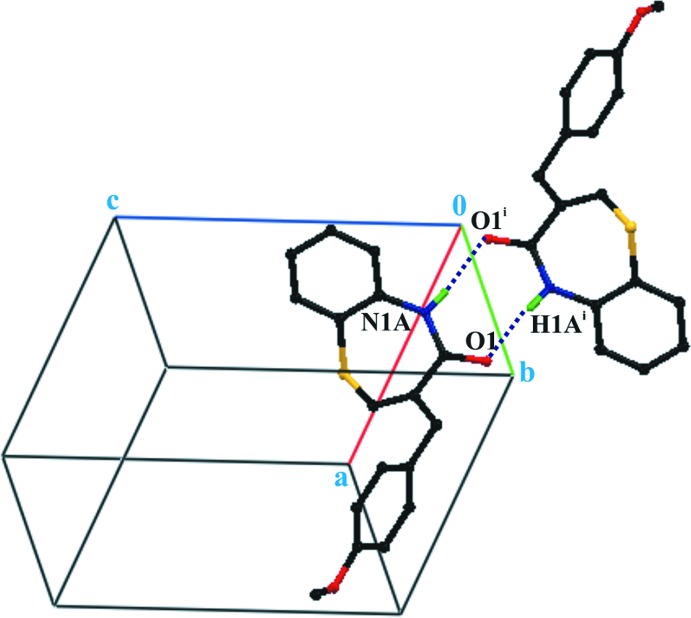
Part of the crystal structure of the title compound showing N—H···O intermolecular hydrogen bonds (dotted lines) generating an R22(8) centrosymmetric dimer. [Symmetry code: (i) -x, 1 - y, -z]. Hydrogen atoms not included in hydrogen bonding are omitted for clarity.

**Fig. 3.**
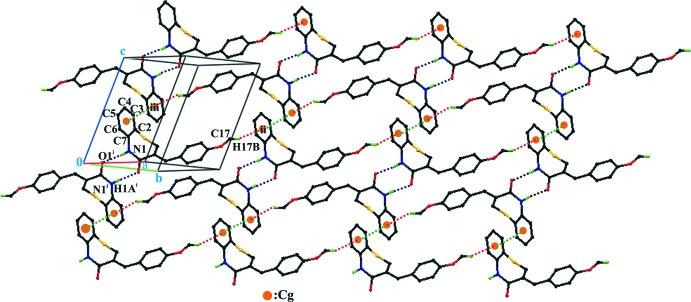
View of three-dimensional supramolecular network. The N—H···O, C—H···π and π—π interactions are shown as blue, red and green dashed lines, respectively. Cg is the centroid of the (C2···C7) benzene ring.[Symmetry code: (i) -x, 1 - y, -z; (ii) 1 + x, 1 + y, z; (iii) -x, 1 - y, 1 - z]. Hydrogen atoms not included in hydrogen bonding are omitted for clarity.

## supplementary crystallographic information

### Comment

The background to the biology of thiazepin derivatives and a related structure have been described recently (Bakthadoss *et al.*, 2013). In view of this biological importance, the crystal structure of the title compound has been carried out and the results are presented here.

Fig. 1. shows a displacement ellipsoid plot of (I), with the atom numbering scheme. The seven membered thiazepine ring (N1/S1/C1/C2/C7/C8/C9) adopts slightly distorted twist-boat conformation as indicated by puckering parameters (Cremer & Pople, 1975) QT = 0.9884 (11) Å, φ_2_ = 357.9 (1)° and φ_3_ = 355.6 (3)°. The dihedral angle between the benzothiazepin ring system and the benzene ring is 65.7 (1) (1)°. The atom O1 deviates by -0.458 (1) Å from the least-squares plane of the thiazepin ring. The sum of angles at N1 atom of the thiazepin ring (359.9^0^) is in accordance with *sp*^2^ hybridization. The geometric parameters of the title molecule agree well with those reported for a similar structure (Bakthadoss *et al.*, 2013).

In the crystal, molecules are linked by N1—H1A···O1 hydrogen bonds into cylic centrosymmetric *R_2_^2^*(8) dimers (Fig. 2 and Table 1). These dimers are further linked by C17—H17B···*Cg*^ii^ (Table 1; Symmetry code:(ii) = *1 + x, 1 + y, z*) hydrogen bonds and π—π interactions between benzothiazepine benzene rings with *Cg*···*Cg*^iii^ = 3.656 (3) Å (Symmetry code:(iii) = *-x, 1 - y, 1 - z*) forming a three-dimensional supramolecular network (Fig. 3; *Cg* is the centroid of the C2–C7 benzene ring).

### Experimental

A mixture of (*Z*)-methyl 2-(bromomethyl)-3-(4-methoxyphenyl)acrylate 2 mmol) and *o*-aminothiophenol (2 mmol) in the presence of potassium *tert*-butoxide (4.8 mmol) in dry THF (10 ml) was stirred at room temperature for 1 h. After the completion of the reaction as indicated by TLC, the reaction mixture was concentrated and the resulting crude mass was diluted with water (20 ml) and extracted with ethyl acetate (3 *x* 20 ml). The organic layer was washed with brine (2 *x* 20 ml) and dried over anhydrous sodium sulfate. It was then concentrated to successfully provide the crude final product ((*Z*)-3-(4-methoxybenzyl)benzo[*b*][1,4]thiazepin-4(5*H*)-one). This was purified by column chromatography on silica gel with ethylacetate/hexane 1:19 as eluent to afford the title compound in good yield (45%). Single crystals suitable for X-ray diffraction were obtained by slow evaporation of an ethylacetate solution at room temperature.

### Refinement

All the H atoms were positioned geometrically and constrained to ride on their parent atom with C—H = 0.93–0.97 Å and N—H = 0.86 Å, and with *U*_iso_(H)=1.5*U*_eq_ for methyl H atoms and 1.2*U*_eq_(C) for other H atoms.

### Crystal data

**Table:**

C_17_H_15_NO_2_S	*Z* = 2
*M**_r_* = 297.36	*F*(000) = 312
Triclinic, *P*1	*D*_x_ = 1.319 Mg m^−^^3^
Hall symbol: -P 1	Mo *K*α radiation, λ = 0.71073 Å
*a* = 7.678 (5) Å	Cell parameters from 5471 reflections
*b* = 9.612 (5) Å	θ = 2.0–32.6°
*c* = 10.860 (5) Å	µ = 0.22 mm^−^^1^
α = 77.208 (5)°	*T* = 293 K
β = 74.117 (4)°	Block, colourless
γ = 81.522 (5)°	0.23 × 0.21 × 0.15 mm
*V* = 748.5 (7) Å^3^	

### Data collection

**Table:**

Bruker APEXII CCD diffractometer	5363 independent reflections
Radiation source: fine-focus sealed tube	3676 reflections with *I* > 2σ(*I*)
Graphite monochromator	*R*_int_ = 0.027
Detector resolution: 10.0 pixels mm^-1^	θ_max_ = 32.6°, θ_min_ = 2.0°
ω scans	*h* = −11→11
Absorption correction: multi-scan (*SADABS*; Sheldrick, 1996)	*k* = −14→14
*T*_min_ = 0.951, *T*_max_ = 0.968	*l* = −15→16
18449 measured reflections	

### Refinement

**Table:**

Refinement on *F*^2^	Primary atom site location: structure-invariant direct methods
Least-squares matrix: full	Secondary atom site location: difference Fourier map
*R*[*F*^2^ > 2σ(*F*^2^)] = 0.045	Hydrogen site location: inferred from neighbouring sites
*wR*(*F*^2^) = 0.134	H-atom parameters constrained
*S* = 1.05	*w* = 1/[σ^2^(*F*_o_^2^) + (0.0608*P*)^2^ + 0.1157*P*] where *P* = (*F*_o_^2^ + 2*F*_c_^2^)/3
5363 reflections	(Δ/σ)_max_ = 0.002
191 parameters	Δρ_max_ = 0.28 e Å^−^^3^
0 restraints	Δρ_min_ = −0.33 e Å^−^^3^

### Special details

**Table:**

Geometry. All e.s.d.'s (except the e.s.d. in the dihedral angle between two l.s. planes) are estimated using the full covariance matrix. The cell e.s.d.'s are taken into account individually in the estimation of e.s.d.'s in distances, angles and torsion angles; correlations between e.s.d.'s in cell parameters are only used when they are defined by crystal symmetry. An approximate (isotropic) treatment of cell e.s.d.'s is used for estimating e.s.d.'s involving l.s. planes.
Refinement. Refinement of *F*^2^ against ALL reflections. The weighted *R*-factor *wR* and goodness of fit *S* are based on *F*^2^, conventional *R*-factors *R* are based on *F*, with *F* set to zero for negative *F*^2^. The threshold expression of *F*^2^ > σ(*F*^2^) is used only for calculating *R*-factors(gt) *etc*. and is not relevant to the choice of reflections for refinement. *R*-factors based on *F*^2^ are statistically about twice as large as those based on *F*, and *R*- factors based on ALL data will be even larger.

### Fractional atomic coordinates and isotropic or equivalent isotropic displacement parameters (Å^2^)

**Table:**

	*x*	*y*	*z*	*U*_iso_*/*U*_eq_	
C1	0.42839 (17)	0.53284 (13)	0.19547 (13)	0.0389 (3)	
H1	0.5174	0.5738	0.2152	0.047*	
C2	0.18042 (19)	0.35576 (13)	0.34697 (13)	0.0404 (3)	
C3	0.1350 (2)	0.29642 (16)	0.47950 (15)	0.0552 (4)	
H3	0.2268	0.2590	0.5217	0.066*	
C4	−0.0434 (3)	0.29249 (19)	0.54865 (16)	0.0639 (5)	
H4	−0.0720	0.2511	0.6368	0.077*	
C5	−0.1794 (2)	0.34965 (18)	0.48779 (16)	0.0595 (4)	
H5	−0.3004	0.3466	0.5347	0.071*	
C6	−0.1378 (2)	0.41157 (16)	0.35764 (14)	0.0475 (3)	
H6	−0.2307	0.4514	0.3172	0.057*	
C7	0.04227 (17)	0.41513 (13)	0.28606 (12)	0.0366 (3)	
C8	0.19209 (16)	0.56421 (13)	0.07296 (12)	0.0344 (2)	
C9	0.32623 (15)	0.61867 (13)	0.12345 (11)	0.0336 (2)	
C10	0.34837 (18)	0.77722 (14)	0.07485 (14)	0.0415 (3)	
H10A	0.3754	0.7968	−0.0196	0.050*	
H10B	0.2336	0.8310	0.1066	0.050*	
C11	0.49498 (17)	0.83050 (13)	0.11561 (13)	0.0377 (3)	
C12	0.45462 (19)	0.90507 (15)	0.21596 (14)	0.0452 (3)	
H12	0.3338	0.9228	0.2601	0.054*	
C13	0.5907 (2)	0.95475 (16)	0.25307 (15)	0.0504 (3)	
H13	0.5607	1.0052	0.3210	0.060*	
C14	0.76966 (19)	0.92861 (14)	0.18852 (15)	0.0456 (3)	
C15	0.81273 (19)	0.85446 (15)	0.08668 (16)	0.0497 (3)	
H15	0.9335	0.8372	0.0423	0.060*	
C16	0.67642 (19)	0.80638 (15)	0.05125 (15)	0.0462 (3)	
H16	0.7066	0.7567	−0.0172	0.055*	
C17	0.8784 (3)	1.0769 (2)	0.2951 (3)	0.0859 (7)	
H17A	0.8026	1.1564	0.2609	0.129*	
H17B	0.9907	1.1094	0.2953	0.129*	
H17C	0.8171	1.0362	0.3825	0.129*	
N1	0.07583 (14)	0.47009 (12)	0.15072 (10)	0.0391 (2)	
H1A	0.0114	0.4382	0.1114	0.047*	
O1	0.18656 (13)	0.60841 (11)	−0.04169 (9)	0.0456 (2)	
O2	0.91500 (16)	0.97189 (13)	0.21607 (13)	0.0668 (3)	
S1	0.41037 (5)	0.34901 (4)	0.25677 (4)	0.05008 (12)	

### Atomic displacement parameters (Å^2^)

**Table:**

	*U*^11^	*U*^22^	*U*^33^	*U*^12^	*U*^13^	*U*^23^
C1	0.0361 (6)	0.0382 (6)	0.0460 (7)	−0.0061 (5)	−0.0172 (5)	−0.0053 (5)
C2	0.0500 (7)	0.0328 (6)	0.0419 (7)	−0.0089 (5)	−0.0177 (6)	−0.0033 (5)
C3	0.0772 (11)	0.0465 (8)	0.0470 (8)	−0.0166 (7)	−0.0288 (8)	0.0044 (6)
C4	0.0900 (13)	0.0610 (9)	0.0383 (8)	−0.0249 (9)	−0.0106 (8)	0.0002 (7)
C5	0.0627 (10)	0.0624 (9)	0.0476 (9)	−0.0190 (8)	0.0023 (7)	−0.0097 (7)
C6	0.0440 (7)	0.0525 (8)	0.0460 (8)	−0.0094 (6)	−0.0091 (6)	−0.0090 (6)
C7	0.0424 (6)	0.0363 (6)	0.0346 (6)	−0.0104 (5)	−0.0117 (5)	−0.0070 (5)
C8	0.0328 (6)	0.0381 (6)	0.0352 (6)	−0.0030 (4)	−0.0122 (5)	−0.0083 (5)
C9	0.0315 (5)	0.0364 (5)	0.0351 (6)	−0.0053 (4)	−0.0111 (5)	−0.0064 (4)
C10	0.0415 (7)	0.0381 (6)	0.0480 (7)	−0.0074 (5)	−0.0196 (6)	−0.0017 (5)
C11	0.0377 (6)	0.0320 (5)	0.0441 (7)	−0.0069 (5)	−0.0140 (5)	−0.0018 (5)
C12	0.0401 (7)	0.0446 (7)	0.0500 (8)	−0.0056 (5)	−0.0076 (6)	−0.0108 (6)
C13	0.0590 (9)	0.0453 (7)	0.0532 (8)	−0.0077 (6)	−0.0170 (7)	−0.0168 (6)
C14	0.0458 (7)	0.0337 (6)	0.0629 (9)	−0.0073 (5)	−0.0253 (6)	−0.0037 (6)
C15	0.0358 (7)	0.0471 (7)	0.0667 (10)	−0.0037 (5)	−0.0121 (6)	−0.0132 (7)
C16	0.0424 (7)	0.0462 (7)	0.0535 (8)	−0.0048 (5)	−0.0114 (6)	−0.0169 (6)
C17	0.0936 (15)	0.0580 (10)	0.138 (2)	−0.0058 (10)	−0.0690 (14)	−0.0361 (12)
N1	0.0399 (5)	0.0479 (6)	0.0349 (5)	−0.0135 (5)	−0.0146 (4)	−0.0064 (4)
O1	0.0453 (5)	0.0596 (6)	0.0358 (5)	−0.0154 (4)	−0.0168 (4)	−0.0019 (4)
O2	0.0591 (7)	0.0568 (6)	0.1027 (10)	−0.0091 (5)	−0.0422 (7)	−0.0231 (6)
S1	0.0448 (2)	0.03683 (18)	0.0684 (3)	0.00032 (13)	−0.02330 (17)	−0.00078 (15)

### Geometric parameters (Å, º)

**Table:**

C1—C9	1.3292 (18)	C10—C11	1.5061 (18)
C1—S1	1.7565 (15)	C10—H10A	0.9700
C1—H1	0.9300	C10—H10B	0.9700
C2—C7	1.3881 (19)	C11—C12	1.3758 (19)
C2—C3	1.392 (2)	C11—C16	1.388 (2)
C2—S1	1.7688 (17)	C12—C13	1.395 (2)
C3—C4	1.372 (3)	C12—H12	0.9300
C3—H3	0.9300	C13—C14	1.375 (2)
C4—C5	1.370 (3)	C13—H13	0.9300
C4—H4	0.9300	C14—O2	1.3721 (17)
C5—C6	1.375 (2)	C14—C15	1.386 (2)
C5—H5	0.9300	C15—C16	1.378 (2)
C6—C7	1.389 (2)	C15—H15	0.9300
C6—H6	0.9300	C16—H16	0.9300
C7—N1	1.4124 (17)	C17—O2	1.415 (2)
C8—O1	1.2337 (16)	C17—H17A	0.9600
C8—N1	1.3474 (16)	C17—H17B	0.9600
C8—C9	1.4935 (16)	C17—H17C	0.9600
C9—C10	1.5170 (18)	N1—H1A	0.8600
			
C9—C1—S1	125.87 (10)	C9—C10—H10B	108.6
C9—C1—H1	117.1	H10A—C10—H10B	107.6
S1—C1—H1	117.1	C12—C11—C16	117.91 (12)
C7—C2—C3	119.01 (14)	C12—C11—C10	121.68 (12)
C7—C2—S1	120.68 (11)	C16—C11—C10	120.41 (12)
C3—C2—S1	120.27 (12)	C11—C12—C13	121.51 (13)
C4—C3—C2	120.83 (15)	C11—C12—H12	119.2
C4—C3—H3	119.6	C13—C12—H12	119.2
C2—C3—H3	119.6	C14—C13—C12	119.54 (13)
C5—C4—C3	119.96 (15)	C14—C13—H13	120.2
C5—C4—H4	120.0	C12—C13—H13	120.2
C3—C4—H4	120.0	O2—C14—C13	124.88 (14)
C4—C5—C6	120.22 (16)	O2—C14—C15	115.39 (13)
C4—C5—H5	119.9	C13—C14—C15	119.73 (12)
C6—C5—H5	119.9	C16—C15—C14	119.91 (13)
C5—C6—C7	120.46 (15)	C16—C15—H15	120.0
C5—C6—H6	119.8	C14—C15—H15	120.0
C7—C6—H6	119.8	C15—C16—C11	121.40 (13)
C2—C7—C6	119.49 (13)	C15—C16—H16	119.3
C2—C7—N1	122.61 (12)	C11—C16—H16	119.3
C6—C7—N1	117.72 (12)	O2—C17—H17A	109.5
O1—C8—N1	119.75 (10)	O2—C17—H17B	109.5
O1—C8—C9	118.99 (11)	H17A—C17—H17B	109.5
N1—C8—C9	121.26 (11)	O2—C17—H17C	109.5
C1—C9—C8	122.57 (11)	H17A—C17—H17C	109.5
C1—C9—C10	122.99 (11)	H17B—C17—H17C	109.5
C8—C9—C10	114.20 (10)	C8—N1—C7	130.70 (10)
C11—C10—C9	114.61 (10)	C8—N1—H1A	114.6
C11—C10—H10A	108.6	C7—N1—H1A	114.6
C9—C10—H10A	108.6	C14—O2—C17	117.51 (14)
C11—C10—H10B	108.6	C1—S1—C2	99.41 (6)
			
C7—C2—C3—C4	1.9 (2)	C9—C10—C11—C16	78.58 (16)
S1—C2—C3—C4	−175.66 (12)	C16—C11—C12—C13	−0.3 (2)
C2—C3—C4—C5	−1.1 (2)	C10—C11—C12—C13	−179.73 (13)
C3—C4—C5—C6	−0.3 (3)	C11—C12—C13—C14	−0.2 (2)
C4—C5—C6—C7	0.9 (2)	C12—C13—C14—O2	180.00 (14)
C3—C2—C7—C6	−1.28 (18)	C12—C13—C14—C15	0.6 (2)
S1—C2—C7—C6	176.27 (10)	O2—C14—C15—C16	−179.96 (13)
C3—C2—C7—N1	−176.30 (11)	C13—C14—C15—C16	−0.5 (2)
S1—C2—C7—N1	1.25 (16)	C14—C15—C16—C11	0.0 (2)
C5—C6—C7—C2	−0.1 (2)	C12—C11—C16—C15	0.4 (2)
C5—C6—C7—N1	175.17 (12)	C10—C11—C16—C15	179.84 (13)
S1—C1—C9—C8	−6.64 (19)	O1—C8—N1—C7	−172.86 (12)
S1—C1—C9—C10	179.39 (10)	C9—C8—N1—C7	6.0 (2)
O1—C8—C9—C1	−134.74 (14)	C2—C7—N1—C8	−51.01 (19)
N1—C8—C9—C1	46.39 (18)	C6—C7—N1—C8	133.88 (14)
O1—C8—C9—C10	39.71 (16)	C13—C14—O2—C17	−14.9 (2)
N1—C8—C9—C10	−139.16 (12)	C15—C14—O2—C17	164.55 (16)
C1—C9—C10—C11	−0.45 (19)	C9—C1—S1—C2	−57.47 (14)
C8—C9—C10—C11	−174.88 (11)	C7—C2—S1—C1	58.98 (11)
C9—C10—C11—C12	−102.00 (15)	C3—C2—S1—C1	−123.50 (11)

### Hydrogen-bond geometry (Å, º)

Cg is the centroid of the C3–C7 benzene ring.

**Table:**

*D*—H···*A*	*D*—H	H···*A*	*D*···*A*	*D*—H···*A*
N1—H1*A*···O1^i^	0.86	2.02	2.860 (2)	167
C17—H17*B*···*Cg*^ii^	0.96	2.96	3.561 (3)	122

Symmetry codes: (i) −*x*, −*y*+1, −*z*; (ii) *x*+1, *y*+1, *z*.
